# Notice to Medical Officers, Etc.

**Published:** 1890-08

**Authors:** 


### NOTICE.

TO THE MEDICAL OFFICERS, ETC., OF THE HOSPITAL DEPARTMENT OF THE LATE CONFEDERATE ARMY AND DEPARTMENT OF TENNESSEE.

Ex-Confederate surgeons, contract physicians, hospital stewards, matrons, and detailed men, who served honorably in the hospital department of the late Confederate Army of Tennessee, under the direction of Dr. S. H. Stout, now residing in Cisco, Texas, are requested to inform him of their present postoffice address. Widows, and nearest of kin of deceased parties above enumerated, are also requested to communicate their postoffice address. Dr. Stout wishes to communicate with them in reference to the preservation of the history of the aforesaid hospital department.

Medical journals and secular newspapers, feeling an interest in the history of the medical service in the late Confederate armies, are requested to copy the above.

